# Status of Health Management Education in India: Past, Present, and Future

**DOI:** 10.3389/fpubh.2018.00375

**Published:** 2019-01-09

**Authors:** Sunitha Kalangi, Harshad Thakur

**Affiliations:** ^1^School of Health Systems Studies, Tata Institute of Social Sciences, Mumbai, India; ^2^Centre for Public health, School of Health Systems Studies, Tata Institute of Social Sciences, Mumbai, India

**Keywords:** health management, health administration, public health, India, hospital administration, management education

## Abstract

This article provides a perspective on the evolution of health management education in India, its current state and the way forward. Health management originated in India in response to the administrative needs of the healthcare system, which is now moving toward institutional care, away from its earlier form of home healthcare. As this field evolved over time, new roles emerged for health management professionals. Several articles have been published in the past describing the state and growth in the field of health management education. This article emphasizes the need to rationalize the sector and shape its future to suit the needs of over a billion people, who use the services of multiple organizations, directly or indirectly in a highly dynamic healthcare environment. We have identified the various challenges that affect the sector today; filling vacant positions, matching jobs with training, and changes in curricula required to achieve good matches. Solutions to address these challenges have also been considered, which in our view could be a way forward in this sector.

## Introduction

Health management in India originated in response to the administrative needs of healthcare providers. It has evolved into a complex specialty with application in many other areas of healthcare. This “hidden career” (1) (it is not an obvious career choice when people think of healthcare services) of health care managers, has different job titles across different organizations, such as health care executive, healthcare administrator, health manager, and public health manager.

The field of Healthcare Management was born as a result of advances in medical sciences, which led to a shift from home-based care to institutional care. The first hospital administration degree program in 1922 at the Marquette University, failed, due to a lack of registrations for the course. The actual process of formal education in hospital administration only started after the publication of Michael Davis's book “Hospital administration, a career” (1).

In India, the existence of hospitals can be traced back to the time of Buddha and Ashoka in the sixth century BC. The health care system has metamorphosed over the centuries, with the invasions by foreigners bringing in hakims and later the European missionaries, leading to the allopathic system of medicine (2). During the British rule in India, several hospitals, medical colleges and dispensaries were established. By the time of independence in 1947, there were 7,400 hospitals with a bed population ratio of 0.24 per 1,000 population (2). The British left a model framework that became the basis for the development of a healthcare structure for the country. However, taking into consideration factors, such as population growth, advent of new diseases and rapid advances in technology, the existing capacities of the system need to be revisited.

The Bhore committee report of 1946 recommended upgrading the health services at all levels and anticipated that the bed population ratio could rise to 1.3/1,000 population in 10 years and to 5.6 in 25 years (3). In order to achieve this, steps taken by the government became the reference points to the way healthcare administration progressed in the country (4). All of this led to the decentralization of health administration, resulting in health administration becoming primarily a responsibility of the state.

To manage the entire system, healthcare management became the bridge between clinical and support functions in the system. Though the original role of a healthcare manager was resource management, over time it was redefined to include other responsibilities. This necessitated the development of systems, practices, interdisciplinary skills, and the creation of capacities to suit the redefined roles (5). The need for an education system, which combined management, social, ethical and psychology related skills along with knowledge of the unique aspects of the healthcare industry, therefore became a necessity.

## Healthcare Management Education in India—History and Overview

A fast-growing economy facing the triple burden of disease, with a population as large as India's, requires a strong public health framework. This can only be made possible with well-qualified public health personnel. Healthcare management is essential in both the private as well as the public health systems, as it plays a crucial role in the successful coordination of multiple resources, diverse people, and complex processes, as well as negotiating with stakeholders to achieve the desired policy objectives and reforms (6).

The High Level Committee Report on Universal Health Coverage (6) in India, 2011, recommended “strengthening health sector management by supporting postgraduate courses in public health and hospital management for the health professionals and health program management for medical, dental, AYUSH (Ayurveda, Yoga, Unani, Sidda and Homeopathy), nursing, and allied health professionals” (6). The committee also recommended the immediate establishment of public health training institutions and to develop strong partnerships with public health management institutions. The report advocated the introduction of a specialized state level health systems management cadre and national level public health service cadres, in order to strengthen the management of the UHC (Universal Health Coverage) system and give greater attention to public health. Such a system would incentivize more people to choose public health management as a career (6).

Traditionally, health management education in India has been offered as part of medical education or as an adjunct to it and was therefore offered only to medical/para-medical professionals. It is a multidisciplinary field that includes aspects of management, medicine, statistics, social sciences, behavioral sciences, finance, operational management, fund raising, law, public policy, and analytics. A recent understanding and acceptance of the multidisciplinary nature of this field has led to the burgeoning of public health schools/institutions, separate from medical educational institutions, which also encourages the enrolment of non-medical graduates. Currently, there are several programs offering courses in different areas of healthcare management.

Hospital administration was the first formal course in the world in the field of healthcare management education. In India, the first masters' degree program in hospital administration was pioneered by AIIMS (All India Institute of Medical Sciences) in 1961 (7). Now there are over 120 colleges offering various diploma, graduate, postgraduate, and doctoral programs in hospital administration.

An example of one such initiative is the establishment of the Public Health Foundation of India in 2006, with a mandate to build public health human resources through the establishment of public health institutions. As a result of their efforts, there are currently five Indian Institutes of Public Health, offering various diploma, certificate, and postgraduate level courses in both general and specialized public health areas (8).

Among the various degree programs offered in public health, a few examples are a Master's in Public Health, MBA (Master's in Business Administration) in Healthcare Management, MD (Doctor of Medicine) in Community Health Administration, MD in Tropical Medicine, and Master's in Health Administration (3). In addition to these, several online/offline certification and postgraduate diploma courses offer specializations in the field. Doctoral programs are offered by institutions, such as the All India Institute of Hygiene and Public Health, the Tata Institute of Social Sciences, and the Indian Institute of Health Management Research.

Currently, 44 institutions offer an MPH (Master's in Public Health) course, of which 26 are privately owned and 18 are public institutions (9). There has been an increase of over 90% in the number of institutions offering an MPH and an increase of 107% in the number of seats available over the last few years Table 1 (9). However, the enrollment rate for these courses, which was 75% in 2010, has fallen to 59% in 2016. Probable reasons for this drop, despite the perceived demand for public health professionals, could be because of the lack of awareness among undergraduates with respect to public health as a profession, limited job opportunities, and the lack of a defined career growth path for such professionals. The geographic distribution of these institutions in India provided in Table 1, also show a lack of connections with regard to the specific needs of the location (9). Only 16% of the institutions are located in the Empowered Action Group (EAG) states, which constitute almost 46% of the country's population and 61% of the poor (10). EAG states are the eight Indian states which are lowest in terms of health indicators and contribute the most to the disease burden of the country (11). It is necessary to reorganize and reinforce the public health system of these states, directly creating the need for public health management professionals.

**Table 1:** Geographical distribution of public health management institutions in India.

**S. No**.	**State**	**Number of Institutions**	**EAG state**
1	Karnataka	8	No
2	Delhi	6	No
3	Maharashtra	5	No
4	Uttar Pradesh	4	Yes
5	Tamil Nadu	4	No
6	Kerala	3	No
7	Chandigarh	2	No
8	Gujarat	2	No
9	Rajasthan	2	Yes
10	Telangana	2	No
11	West Bengal	2	No
12	Himachal Pradesh	1	No
13	Nagaland	1	No
14	Odisha	1	Yes
15	Puducherry	1	No
	Total	44	

## The Way Forward

The healthcare industry in India is rapidly expanding, with multi-million-dollar investments made by various national and international agencies, the pharmaceutical sector, central and state governments, and developmental partners. The health sector is projected to grow at the rate of 23% per annum to a record US$77 billion industry by 2022, according to Yes Bank and an industry body report published in November 2009 (12). In order to respond to this growth effectively, there is a need to create human resource capacities in the areas of public health management and hospital administration. Though the process of building human resource capacities in healthcare management has already started, there is still scope for a lot of learning and education, in order to achieve the objective of a strong healthcare management workforce. Table 2 presents important tasks to be considered in shaping the future of healthcare management education.

**Table 2:** The way ahead for health management education in India.

**Area of concern**	**Need and recommendations**
Quality of education	Need a central regulating body for assessment of the quality of the programs, and monitoring of the institutions offering the programs An accrediting body would help guide the process of standardization of the programs.
Curriculum design	Standardization of the curriculum Allied skill development Increased industry exposure as part of the curriculum Limited flexibility in curriculum design to allow local context
Faculty development	Capacity building to meet the demand of faculty in terms of numbers and quality Need for a multi-disciplinary faculty To introduce more doctoral programs to build capacities
Assessment of demand and supply	Need for a realistic assessment of the demand for the various specializations in the profession Need to assess the available pool of professionals and analyze the demand and supply rationally
Continuous education	Courses to be made available for working professionals to update themselves in the latest developments in the industry and develop new skill sets wherever relevant
Creation of career paths	Bridge the disconnect between demand and supply of professionals existing in this field Create clear career paths for the professional Create awareness regarding the various career opportunities available for professionals in the field.

### Quality of Education and Accreditation

The lack of standardized curricula in various institutions offering health management programs and the absence of adequate regulatory and quality control mechanisms, have led to a large variation in the quality of the outgoing product (13). In the USA, a separate body, the Association of University Programs for Hospital Administration (AUPHA) exists to represent the various educational programs in health administration. AUPHA also started the formal accreditation process for the various educational programs, which is currently being undertaken by a separate organization—the Commission on Accreditation of Healthcare Management Education (CAHME).

In India, accreditation of educational institutions is a relatively new concept. Currently, professional councils, such as the All India Council of Technical Education (AICTE), the University Grants Commission (UGC), and the Medical Council of India (MCI) are responsible for recognizing the various health management courses in the country. The National Board of Accreditation (NBA), which was established by AICTE, and the National Assessment and Accreditation Council (NAAC), established by the UGC, provide accreditation at the institution level and have currently constituted expert groups to develop a program level accreditation process in the country. The institutional level accreditation that is available currently may not be sufficient to satisfy the need for dynamic contextual validation of the courses required for industry specific programs like healthcare management.

### Curriculum Design

There is a need to revisit the curriculum and standardize it to ensure uniformity of the minimum core competencies among all the graduates. It must be in line with modern education systems, as well as the technological advances in the sector. In addition, the curriculum should allow for variations to accommodate the healthcare needs of a highly diverse population in different regions in the country.

For example, as seen from Table 3, the highest death rate from Ischemic Heart Disease among the states, is 12 times the lowest rate, while the death rate from Chronic Obstructive Pulmonary Disease is nine times the lowest rate across the states of India (11). Similarly, there is a large urban and rural divide in terms of health status, as well as health resource distribution in the country. For example, the infant mortality rate is 44/1,000 in rural areas as compared to 27/1,000 in urban areas while the mortality ratio at all ages is 7.6 in rural areas compared to 5.6 in urban areas (14). Seventy percent (70%) of the health infrastructure, medical manpower and other resources are concentrated in urban areas, which account for <70% of the population (15). This kind of diversity requires a public health strategy and system to suit the local context; and thus, has an implication on the curriculum used to impart training to the public health experts managing the system. The capabilities and training needs of a public health management professional working in a natural disaster-prone area would be different to those of one working in a region more affected by lifestyle diseases. These differences could thereby determine the curricular design of the various specializations in the field.

**Table 3:** Death rate per 100,000 due to individual causes in India.

**Cause of death**	**National average**	**Lowest**		**Highest**	
		**Rate**	**State**	**Rate**	**State**
Ischemic heart disease	132	27	Mizoram	261	Punjab
COPD (Chronic Obstructive Pulmonary Disease)	64	22	Meghalaya, Delhi	111	Rajasthan
Diarrheal diseases	59	11	Delhi	129	Odisha
Tuberculosis	33	8	Kerala	58	Uttar Pradesh

A joint working group of the United Kingdom (UK) and India was formed to create a model guideline for the MPH curriculum (16). This document defines the core and elective requirements of the course, taking into account the development of the skills, competencies, knowledge, and values required for a public health professional. This document, in addition to local and social considerations, can help serve as a useful guide for institutions to revisit their curricula, and to line them up with the needs of the country.

The teaching/learning methods used also need to be re-examined. In most institutions, the majority of learning is done through didactic lectures. There is a need for a more inquiry-driven form of learning, mixed with considerable exposure to the actual health scene in the country, including public health programs, public and private hospitals, rural health programs, health tourism, telemedicine, pharmaceuticals, hospital planning, healthcare consultancies, health technology assessment, and the medical devices industry. This will not only help students understand the various opportunities available but will also help them to form a point of view of the various stakeholders in the industry.

### Faculty Development

The Lancet Commission, “Health professionals for a new century: transforming education to strengthen health systems in an interdependent world,” brought into light a perspective shared by leaders from the industry, as well as academics, to make public health education free from the silos of individual professions (17). It follows that a multidisciplinary approach should be used for teaching, to prepare students for the reality of practice. This kind of trans-disciplinary approach has also been advocated by two reports from the Institute of Medicine in 2002 (18) and 2003 (19).

Currently, faculty for public health management programs constitute members from multiple fields. However, courses offered as an adjunct to medical colleges are taught predominantly by medical faculty. There is a shortage of suitably trained/experienced faculty for these programs. Additional capacity in terms of the number and a better quality faculty can be created by introducing more doctoral level programs in this area (20).

### Assessment of Demand and Supply

**Figure 1:**
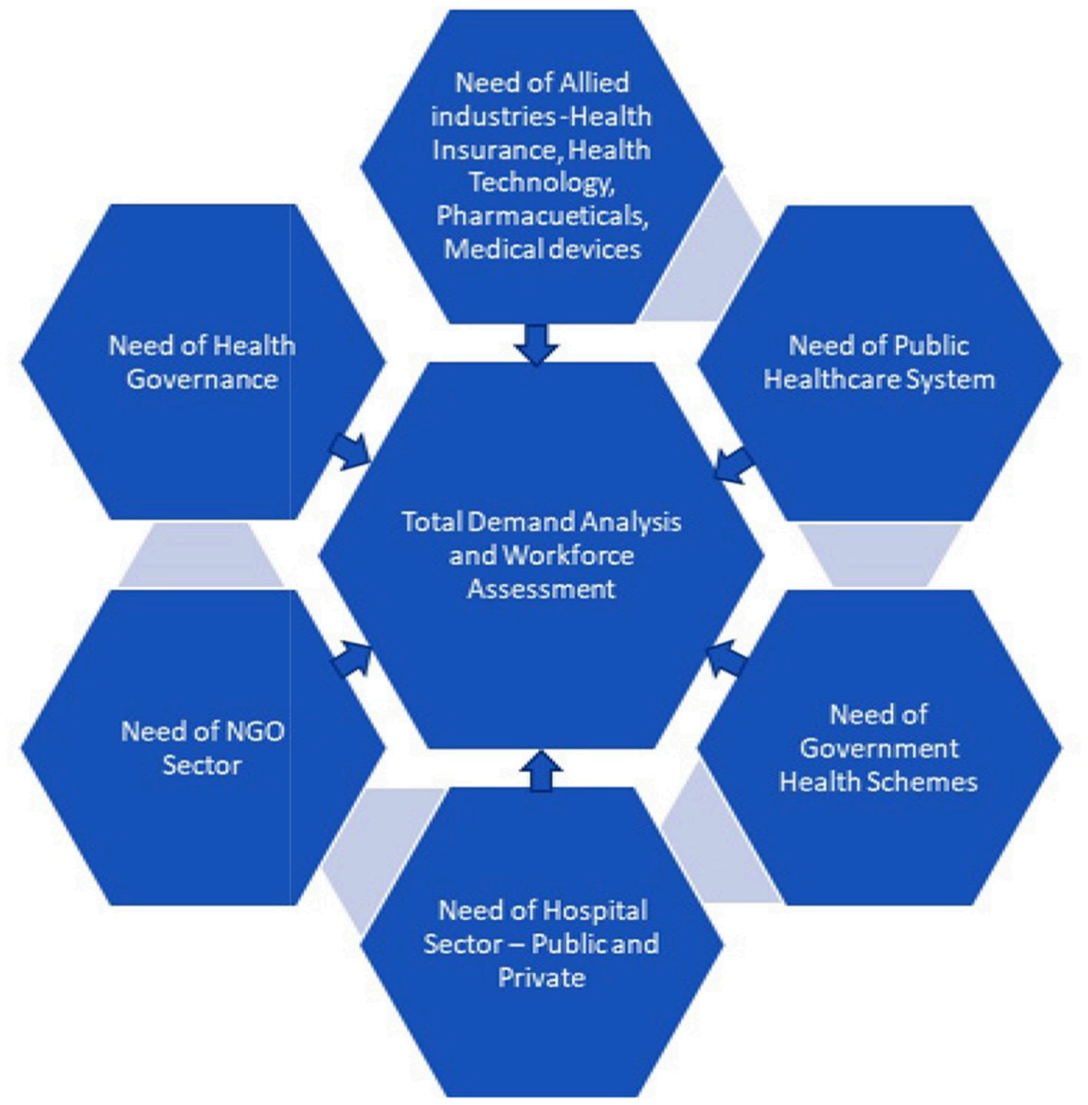
Demand and workforce assessment—a snapshot.

Currently, no valid assessment is available of the number and types of healthcare professionals required to serve the industry. This is critical information, as it forms the basis for estimating the capacities required for the future; both in terms of quantity and the type of professionals required. As depicted in Figure 1, a demand analysis could be made based on a detailed work force assessment that takes into consideration the requirements of the following: the state and district level agencies in the public healthcare system, various governmental health schemes, requirements of private/public hospital sector/non-governmental organizations sector, allied areas of healthcare management, such as health insurance, health technology, governance, and pharmaceuticals.

### Continuing Education

The rapidly changing technologies used in the healthcare sector require managers to have the capacity to cope with the dynamic nature of the field. As Charles Darwin said, “It is not the strongest of the species that survive nor the most intelligent, but rather the one that is most responsive to change” (21). Continuous education is therefore essential to keep abreast with the changes in the field and which will enable professionals to make the necessary adjustments to cope with the changes in their respective work environments (21).

### Creation of Career Paths

There is a general agreement on the dearth of qualified healthcare management professionals in the industry, implying a demand for the same. However, data shows that there aren't enough enrollments for the seats available. This suggests that the focus should not be on increasing the capacity of the system, instead on streamlining it to attract more students to choose healthcare management as a career path (22). Clear career options and growth prospects along with commensurate compensation mechanisms for these professionals (23) are currently the most important need.

Overall, Table 2 summarizes the way forward and can even be considered as a recommendation for the way forward.

## Conclusion

Several articles have been published in the past, describing the status and structure of the field of healthcare management education in India. The challenges lie not in the capacity of the education system but in the structure, content, quality, and the distribution of the programs offering training in healthcare management. There is a need for a certain level of consistency among the programs with respect to the structure and content of the curriculum, in order to ensure the inclusion of a base set of competencies for all graduates in this field. A comprehensive needs assessment of this sector is therefore required, not only to rationalize the development of specialty courses to suit the requirement of the sector, but also to adequately streamline the geographic distribution of the institutions as well as the structure of courses, to suit the context. A regulatory/accreditation agency, to guide and monitor the programs along similar lines of AUPHA, would go a long way toward quality control of these programs. It would also help build a network of professionals to exchange information and bridge the gap between academics and industry. It would also fill the gap of continuing education that is lacking in the current system. There is a need to focus on methods to increase enrolment to these courses, by increasing awareness and developing better career paths for these professionals. Revisiting the education system in this sector would help ensure that it is in line with the dynamic needs of a complex healthcare system, of a large country, with a population of more than a billion people. A higher degree of consistency across programs and defining the base set of competencies for all graduates in the field, would help them regardless of which management positions they assume.

## Author Contributions

All authors listed have made a substantial, direct and intellectual contribution to the work, and approved it for publication.

### Conflict of Interest Statement

The authors declare that the research was conducted in the absence of any commercial or financial relationships that could be construed as a potential conflict of interest.
